# Red Wine Lees Valorization for Phenolic Compounds Recovery and Characterization Using Resin Adsorption

**DOI:** 10.3390/molecules31213929

**Published:** 2026-11-01

**Authors:** Antonis Vasilopoulos, Varvara Sygouni, Christina Vasiliki Lazaratou, Athanasia G. Tekerlekopoulou, Dimitris V. Vayenas

**Affiliations:** 1 Department of Chemical Engineering, University of Patras, 26504 Patras, Greece; vasilopantonis@chemeng.upatras.gr (A.V.); dvagenas@upatras.gr (D.V.V.); 2 Department of Sustainable Agriculture, University of Patras, 30100 Agrinio, Greece; atekerle@upatras.gr; 3 Foundation for Research and Technology—Hellas, Institute of Chemical Engineering Sciences, FORTH/ICE-HT, 26504 Patras, Greece

**Keywords:** wine lees, phenolic compounds, resin adsorption, XAD-16 resin, LC-HRMS profiling

## Abstract

Wine lees are a phenolic-rich winery by-product whose liquid fraction remains underexploited. This study investigated the direct recovery of phenolic compounds from the liquid fraction of red wine lees (LfWL) using the non-ionic Amberlite XAD-16 resin through batch and fixed-bed adsorption, followed by sequential desorption and LC-HRMS profiling. Batch adsorption experiments were conducted at resin dosages ranging from 20 to 120 g/L. The highest adsorption yield (89.9%) was achieved at 120 g/L, whereas the highest experimental adsorption capacity (123.5 mg SAE/g) was obtained at 20 g/L. Adsorption kinetics were better described by the pseudo-second-order model, whereas the non-linear Langmuir model provided the best equilibrium fit, with a model-estimated maximum adsorption capacity of 304.34 mg SAE/g. Phenolics adsorption was favored at the natural pH of the LfWL, while the thermodynamic analysis indicated an exothermic and thermodynamically favorable process. Two different fixed-bed columns were evaluated under continuous flow conditions. The configuration with larger dimensions (LFB-200) treated an undiluted LfWL, reaching an adsorption efficiency of 36.59% with a breakthrough profile extending beyond 1400 min. Sequential elution with water followed by a 50% (*v*/*v*) ethanol/water mixture resulted in desorption yields of 88.96% and 79.54% for the small and large columns, respectively. LC-HRMS enabled the putative annotation of 20 phenolic compounds and revealed compound and class-dependent elution profiles. These results support resin adsorption as a promising route for direct phenolic recovery from liquid wine lees fractions.

## 1. Introduction

The global winemaking industry represents an important sector of agricultural production but also generates substantial amounts of by-products, including grape stalks, grape pomace, wine lees and winery wastewater [1]. Currently, these winery wastes are managed through incineration and landfilling without considering the environmental risks they pose due to their acidic pH, high organic load, and content of potentially phytotoxic compounds [2,3]. According to the International Organisation of Vine and Wine (OIV), in 2025, an estimated 227 million hectoliters (mhl) of wine were produced globally, representing a slight increase of 0.6% compared with 2024, while the European Union produced 136 mhl [4]. One of the main winery by-products is wine lees, which account for 14–25% of total wastes and 2–6% of the wine produced [5,6]. According to European Union Regulation No. 1308/2013, wine lees are defined as “the residue accumulating in vessels containing wine after fermentation, during storage or after treatment as well as the residue obtained from filtering or centrifuging this product”. Furthermore, the mechanical pressing of lees to recover liquid intended for human consumption is prohibited; liquid must be withdrawn under the supervision of competent authorities and in compliance with environmental legislation. Delivery to distillation constitutes one of the available disposal routes and may be made compulsory by individual Member States [7].

Wine lees mainly consist of dead yeast cells, bacteria and other residues, and their composition is influenced by grape variety, fermentation conditions, place of cultivation and aging time [8]. Due to the high oxygen demand of the organic compounds found in wine lees, they are considered a harmful by-product for the environment and soil if they are discharged into landfills [9]. Moreover, high concentrations of phenolic compounds possess phytotoxic properties that can disrupt plant transpiration rates and overall survival [10]. To mitigate these negative effects, the valorization of wine lees can be considered a sustainable approach within the circular economy perspective, aiming to recover high value-added biocompounds such as polyphenols [11]. Specifically, during winemaking, phenolic compounds are transferred from grapes to wine and then into lees. These recovered polyphenols are of significant interest to the pharmaceutical, cosmetic and nutraceutical industries because of their antioxidant, anti-inflammatory and anti-cancer properties, which are associated with the prevention of chronic diseases and certain types of cancer [11].

Wine lees consist of solid and liquid phases, with the latter being mainly processed for alcohol recovery and tartaric acid isolation, or otherwise managed as an organic residue [12]. Several valorization strategies have been reported, including the recovery of phenolic compounds from wine lees through solvent or microwave-assisted extraction, sometimes followed by spray drying or membrane fractionation [13,14,15]. Recent membrane-based approaches have included an initial extraction step involving hydroalcoholic extraction of white wine lees or aqueous extraction of red wine lees followed by membrane separation [16,17]. Unlike these extraction-based schemes, the present approach employs direct processing of the liquid phase, eliminating the need for a separate solid–liquid extraction step and enabling the recovery of phenolic compounds already dissolved in the hydroalcoholic phase. The distinct chemical profiles previously observed for the solid and liquid fractions of wine lees further support their separate valorization [12].

Adsorption onto non-ionic polymeric resins is a promising approach for the recovery and concentration of phenolic compounds from complex liquid matrices. These resins have been applied to phenolic recovery from several agro-industrial by-products, including pomegranate residues [18], apple pomace [19], and olive oil washing wastewater [20]. More recently, five non-ionic polymeric resins were evaluated for polyphenol recovery from white wine lees extracts [13], whereas batch and fixed-bed adsorption were investigated for wine vinasses, which constitute a different post-distillation matrix [21]. Moreover, UHPLC-HRMS has recently been employed to provide compound-level characterization of the phenolic compounds in white wine lees [8]. Nevertheless, information remains limited on an integrated process combining batch adsorption, continuous fixed-bed operation, breakthrough curve modeling, sequential desorption, and LC-HRMS profiling of the recovered fraction.

In this study, the liquid fraction of red wine lees (LfWL) was directly processed using the non-ionic Amberlite XAD-16 resin, without an additional solid–liquid extraction step. To the best of our knowledge, limited research has been conducted on the direct adsorption treatment of the liquid fraction of wine lees. Therefore, batch experiments were first performed to characterize and optimize the adsorption behavior by investigating the effects of resin dosage, contact time, pH and temperature, while kinetic, equilibrium and thermodynamic models were applied to further describe the adsorption process. Subsequently, continuous fixed-bed experiments were conducted to assess the applicability of the adsorption approach under flow conditions, and the experimental breakthrough curves were fitted using the Thomas, Yoon–Nelson, and Clark models. The adsorbed phenolic compounds were then recovered through sequential desorption, using water followed by a 50% *v*/*v* ethanol–water solution. Finally, LC-HRMS/MS analysis was employed to characterize the phenolic compounds present in the recovered fractions. A combined batch, fixed-bed, desorption, and LC-HRMS/MS approach was used to evaluate direct liquid fraction processing as a potential route for the valorization of red wine lees.

## 2. Results and Discussion

### 2.1. Batch Adsorption Experiments

Batch adsorption experiments were conducted using the liquid fraction of the red wine lees (LfWL) obtained after centrifugation and vacuum filtration. These experiments were carried out to examine a range of parameters, such as the effect of resin dosage (adsorbent), pH adjustment, and thermodynamic temperature variations, to examine the adsorption kinetics and construct the adsorption isotherms.

#### 2.1.1. Effect of Adsorbent Dosage

**Figure 1:**
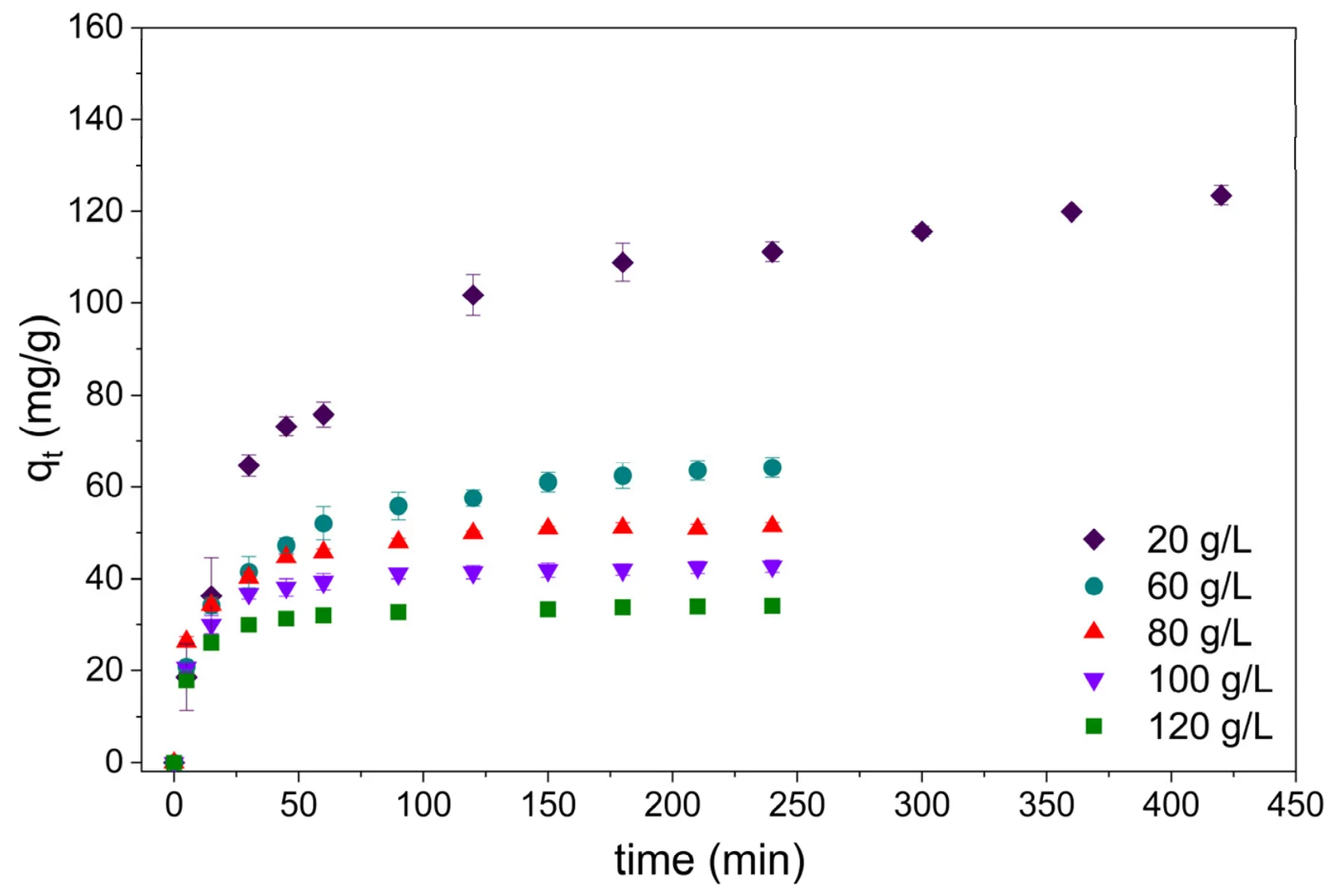
Experimental results of batch adsorption of phenolics from LfWL using different adsorbent concentrations.

Initially, the effect of resin dosage was investigated by varying the XAD-16 dosage from 20 to 120 g/L while maintaining the initial TPC of the LfWL (4709.8 mg SAE/L). The use of various resin dosages resulted in different equilibrium concentrations. Increasing the mass of the adsorbent accelerated the equilibrium state and, as expected, resulted in higher phenolic adsorption yields while reducing resin adsorption capacity ($q_{e}$) (Figure 1). In the case of higher resin dosages (60–120 g/L), equilibrium was reached within 4 h of the experiment, achieving a TPC adsorption yield between 81.0% and 89.9%, and the adsorption capacity decreased from 64.2 mg SAE/g to 34.1 mg SAE/g, respectively. In contrast, at the 20 g/L dosage, only 52.4% of the initial TPC was adsorbed within 7 h, demonstrating a significantly higher adsorption capacity of 123.5 mg SAE/g. This may be attributed to the excess of available active sites per unit surface area of the adsorbent at higher resin dosages. Moreover, in previous studies, it was observed that the addition of a high mass of the adsorbent resulted in particle agglomeration, causing an overlapping of the active binding sites and thereby reducing the total surface area accessible to the solutes [22,23].

Regarding process kinetics, the time required to reach equilibrium in the LfWL samples (4 to 7 h) aligns with the findings of Zadeh et al., 2022, who reported that XAD-16 resin required 6 to 8 h to attain steady-state adsorption when treating hazelnut skin extract [24]. They also calculated an adsorption capacity of 41.15 mg/g with an initial TPC of 5 g GAE/L and a resin dosage of 20 g/L.

Furthermore, Amberlite XAD-16 exhibited a notable phenolics adsorption performance for the LfWL. At a resin dosage of 60 g/L, the calculated equilibrium capacity in the present study reached 64.2 mg SAE/g, with adsorption recovery yielding 81% (adsorption yield (%)), while the capacity and recovery yields reported by Soto et al., 2012 [25], for winery by-products using XAD-16HP were ~2.3 mg/g and 80.2%, respectively. Furthermore, this performance remains highly competitive against other heavy agro-industrial residues; for comparison, Kodjapashis et al., 2023, reported an equilibrium capacity near 60 mg/g for an olive leaf phenolic extract onto Amberlite XAD16N at a 20 g/L dosage [26], whereas the LfWL system achieved 123.5 mg SAE/g under the same adsorbent load. In general, the adsorption capacity of a resin depends on both the properties of the resin itself (i.e., its surface area, pore size, and particle structure) and the nature of the target molecules. The phenolic compounds present in the LfWL could be adsorbed through physical mechanisms thanks to a combination of weak van der Waals forces, hydrogen bonding, and π-π interactions between the aromatic rings of the phenolics and the benzene rings of the resin [24,27]. Variations in the reported parameter values in the literature can be attributed to the method used to express TPC, e.g., gallic acid equivalents or syringic acid—as used in this work—and the polyphenols adsorbed in each case [26].

#### 2.1.2. Adsorption Kinetics

The experimental data obtained from the batch experiments with various adsorbent concentrations were fitted using the linear and non-linear forms of the pseudo-first-order (PFO) and pseudo-second-order (PSO) kinetic models. Based on the analysis, the non-linear PSO model provides the most accurate description of the process, as indicated by the high R^2^ values (0.990–0.999) (Table 1 and Table 2). There is also a close correspondence between $q_{e,cal}$ and $q_{e,exp}$, alongside the lowest RMSE values. On the other hand, despite the relatively high correlation coefficients in both the linear (0.914–0.978) and non-linear (0.943–0.981) forms, the PFO model failed to predict the accuracy of the kinetic behavior, compared to the fitting parameters of the PSO model. This failure is further underscored by the RMSE values, where the non-linear PFO model exhibited higher RMSE values (1.53–6.00 mg/g) across all resin dosages compared to the errors of the non-linear PSO model (0.14–3.05 mg/g), which were lower.

These remarks suggest that the rate of phenolic adsorption may be governed by multiple diffusion steps [27], not only by diffusion through the interface [28]. The pseudo-second-order kinetics that fit the interaction of phenolic compounds with the XAD-16 resin indicate that the rate is primarily dependent on the resin adsorption capacity and not the adsorbate concentration [28]. Although PSO has traditionally been associated with chemisorption [29], goodness of fit alone cannot establish the nature of adsorbate–adsorbent interactions or identify the rate-controlling mechanism. Detailed thermodynamic analysis and desorption experiments can provide complementary details of the process and help distinguish chemical from physical sorption.

In the work by Gao et al. [30], the authors examined the adsorption of cranberry pomace phenols, specifically procyanidins, on XAD16N resin. They observed that the PSO model fitted their experimental results better, with a $k_{2}$ rate constant of 7.9 × 10^−5^ g/(mg·min). This value is one order of magnitude lower than the one estimated in the present study, 2.1 × 10^−4^ g/(mg·min). Dong et al. [31] examined the adsorption of flavonoids onto XAD16 resin from *Glycyrrhiza glabra* L. leaf using a resin dosage of 50 g/L and an equilibrium spanning between 30 and 1080 min. They also identified the PSO model as the most favorable for describing the process, with a $k_{2}$ value of 4.13 × 10^−2^ g/(mg·min).

Furthermore, Kodjapashis et al. [26] studied the adsorption of olive leaf extract phenolic compounds onto XAD16N resin and reported a $k_{2}$ value of 7.09 × 10^−4^ g/(mg·min), which is highly comparable to the findings of this study (2.1 × 10^−4^ g/(mg·min)).

In conclusion, most studies identify the PSO kinetic model as the most appropriate to describe the adsorption process. The observed variations in rate constants and adsorption capacities across studies may also be attributed to the method used to express TPC (GAE or SAE). Syringic acid is characterized by its methoxylated aromatic structure and shares structural features with certain phenolic constituents that are potentially present in wine lees. However, the heterogeneity of these multicomponent waste matrices, including variations in phenolic composition, molecular size and interactions with other organic constituents, is likely to account for differences in adsorption performance between studies. Different phenolic fractions may exhibit varying affinities for the resin. For example, in this study, high-molecular-weight phenols may exist, making the time to equilibrium longer than in other studies. This behavior occurs because larger phenolic molecules may not be able to easily access all the available adsorption sites within the resin’s microporous structure. However, if a larger molecule successfully binds to an active site, it contributes a higher adsorbed mass per single occupied site, leading to higher calculated adsorption capacity values [26].

#### 2.1.3. Adsorption Isotherms

The equilibrium data collected from the five batch experiments were used to obtain adsorption isotherms. Two models were examined in both linear and non-linear forms: the Langmuir model and the Freundlich isotherm. The calculated parameters are summarized in Table 3 and Table S1.

The non-linear forms of both the Langmuir and Freundlich models (Figure S1) exhibited higher correlation coefficients and lower RMSE values than the linear approaches. The Langmuir model outperformed the Freundlich model, displaying a higher R^2^ value (0.9941 and 0.9923, respectively) and a lower RMSE (3.151 mg/g and 3.587 mg/g). Under the linear approach, Langmuir estimated a theoretical maximum adsorption capacity ($q_{max}$) of 404.86 mg SAE/g, which is higher than the non-linear estimate (304.34 mg SAE/g). Both values indicate a high adsorption capacity of the resin, which could be attributed to extrapolation of the models beyond the experimentally investigated equilibrium range. Therefore, the use of a wider range of adsorbent dosages or varying initial TPC values of the LfWL under a constant resin dosage would be useful to define the saturation range of the resin. According to the literature, non-linear regression is preferable to linearized isotherm fitting because transformation of the Langmuir or Freundlich equations changes the error structure and may disproportionately weight particular concentration regions [32]. In conclusion, the non-linear Langmuir model provided a slightly better fit to the equilibrium data than the non-linear Freundlich model according to the combined R^2^ and RMSE values.

Different isotherm models have also been reported for phenolic adsorption onto XAD-16 resin. Langmuir-type behavior has been observed in some phenolic-rich agro-industrial wastes, whereas the Freundlich and Liu isotherm models have provided a better description in systems displaying greater heterogeneity [5,33,34,35,36]. For example, Yang et al. reported Freundlich-type adsorption for phenolic compounds recovered from adlay bran using macroporous resins [33]. More recently, Franzen Ramos et al. [34] found that adsorption of polyphenols from coffee wastewater could be described by both the Freundlich and Liu equations, reflecting the complexity of adsorption from multicomponent wastes. The variation between studies is consistent with differences in resin properties, feed composition, and competition from non-phenolic substances. The satisfactory performance of the Freundlich isotherm indicates that energetic heterogeneity and differences in compound affinity may still contribute to adsorption behavior. For these reasons, it would be very interesting to apply alternative isotherm models that combine Langmuir–Freundlich behavior, such as the Sips isotherm [37].

#### 2.1.4. Effect of pH

**Figure 2:**
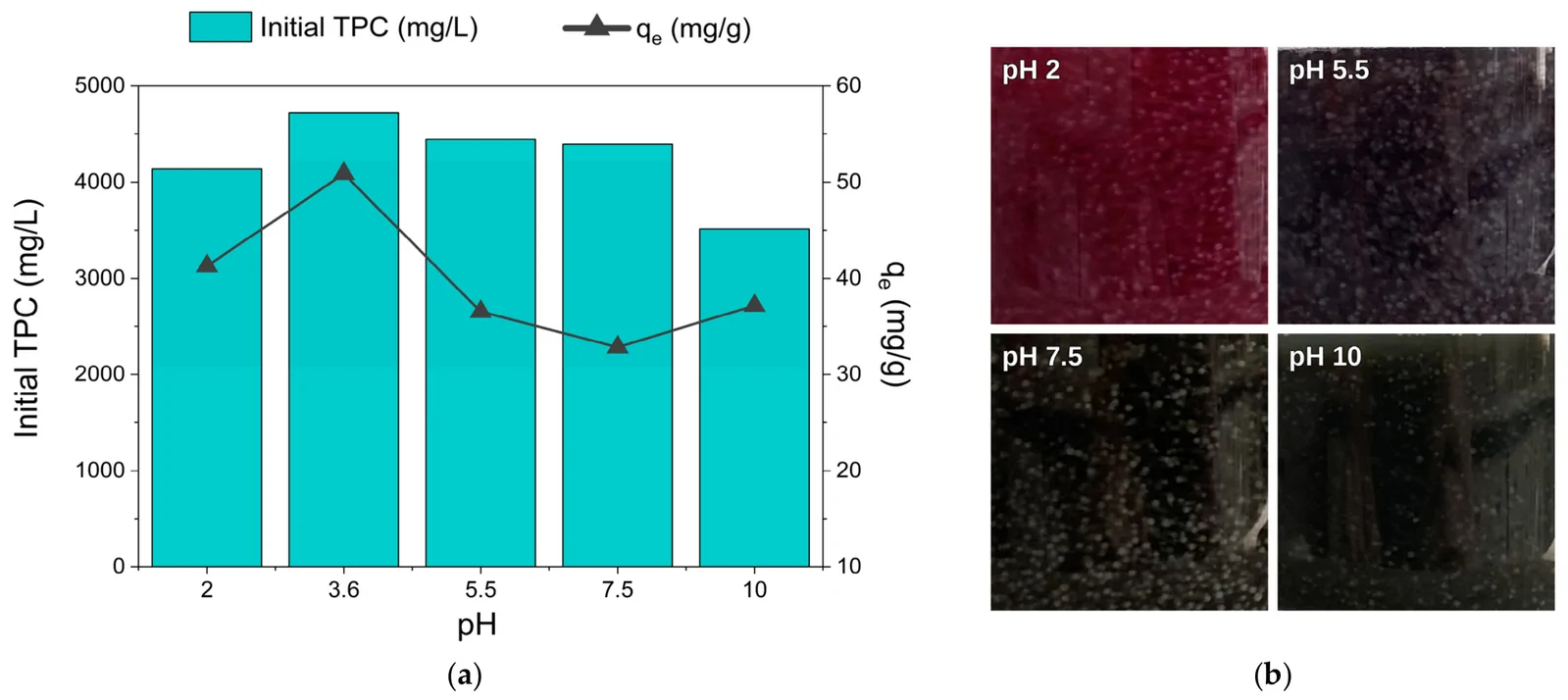
(**a**) Effect of solution pH on the initial TPC and the corresponding equilibrium adsorption capacity (q_e_). (**b**) Visual observation of the color change of LfWL samples after pH adjustment (ranging from 2.0 to 10.0).

The effect of pH (values 2.0, 3.6, 5.5, 7.5, 10.0) is demonstrated in Figure 2a. For this purpose, an initial resin dosage of 80 g/L was used in all five experiments. Immediately after the pH adjustment and prior to the addition of the resin, the initial TPC was measured to find out whether the pH modification induced any precipitation of the phenolic compounds (TPC). Upon increasing the pH from 2.0 to 3.6, the adsorption capacity increased slightly, while further increases in pH led to a decrease in the adsorbed amount. A similar trend was observed during pH adjustment of white tea flavonoids [38]. Moreover, the decrease in TPC observed at pH 2 compared with an untreated LfWL (pH 3.6) may be attributed to the increased protonation of carboxyl groups under more acidic conditions [39]. These changes may influence the solubility and interactions between phenolic compounds and other components of the lees matrix (e.g., endogenous yeast proteins), potentially promoting the formation and precipitation of insoluble protein–phenolic complexes below the protein isoelectric point [40] and consequently reducing the concentration of soluble phenolics detected by the Folin–Ciocalteu method. The optimum capacity occurs at the natural pH of the LfWL (3.6). While adjusting the pH of the LfWL samples, a distinct color change was observed (Figure 2b). Specifically, after adjusting the pH from 3.6 to 2.0, making it more acidic, the color changed from red to pink. Likewise, adjusting to neutral pH caused a transition to a light green color, while in an alkaline environment, the color turned dark green. Studies involving anthocyanin-rich extracts from wine lees have demonstrated that pigment stability can vary with pH [41,42]. These color changes were accompanied by a decrease (25% at alkaline pH) in the initial TPC, as shown in Figure 2a.

Adjusting the pH of the LfWL to 10.0 caused an immediate decrease in the initial TPC from approximately 4700 mg/L to 3512 mg/L before the addition of resin. According to Pasquet et al. [43], exposing phenolic compounds to an alkaline environment in the presence of atmospheric oxygen triggers rapid degradation. Under these conditions, the hydroxyl groups of phenolics are converted into phenolate anions, which are oxidized by atmospheric oxygen to form highly reactive semiquinone radicals and finally quinones. This decrease in initial phenolic concentration may also take place because of monomerization of phenolic compounds during alkalization [44]. This phenomenon may explain the pronounced decrease in the initial TPC observed at pH 10.0. Since these oxidation products exhibit a lower reducing capacity toward the Folin–Ciocalteu reagent [43], their formation can result in a lower measured TPC. Therefore, the higher apparent adsorption capacity determined at pH 10.0 should not be attributed exclusively to enhanced adsorption, as alkaline oxidation of phenolic compounds also contributes to the observed decrease in TPC.

#### 2.1.5. Thermodynamic Study

**Figure 3:**
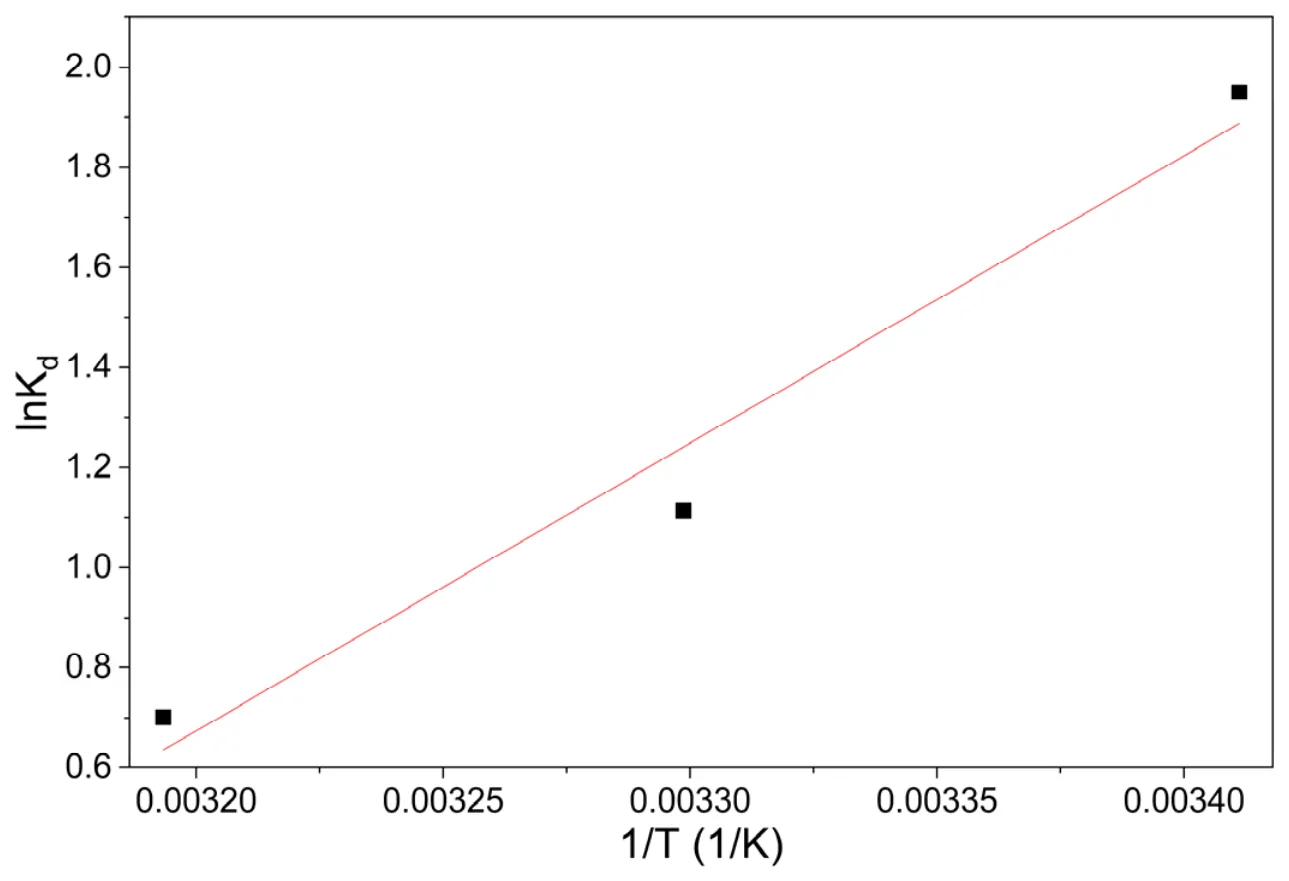
Van’t Hoff plot for the adsorption of phenolic compounds from the LfWL onto XAD-16 resin.

The effect of temperature on the adsorption of phenolic compounds from the LfWL was studied in the temperature range from 20 °C to 40 °C. Figure 3 illustrates the Van’t Hoff plot of lnKd versus 1/T with a coefficient of determination (R^2^) of 0.9634. From this linear regression, the thermodynamic parameters, including standard enthalpy change (ΔH^0^), standard Gibbs energy (ΔG^0^) and standard entropy change (ΔS^0^), were estimated (Table 4).

The negative enthalpy value (−47.78 kJ/mol) indicates an exothermic process between the targeted molecules and the surface of the resin during adsorption. Moreover, the negative free energy change (ΔG^0^) suggests that the adsorption onto the XAD-16 resin was spontaneous. The decrease in the absolute value of ΔG^0^ with increasing temperature further indicates that the adsorption became less favorable at higher temperatures, which is aligned with the exothermic nature of the system. Lastly, the negative entropy change (ΔS^0^) suggests a reduction in randomness at the solid–liquid interface [45,46].

### 2.2. Column Adsorption of the LfWL

**Figure 4:**
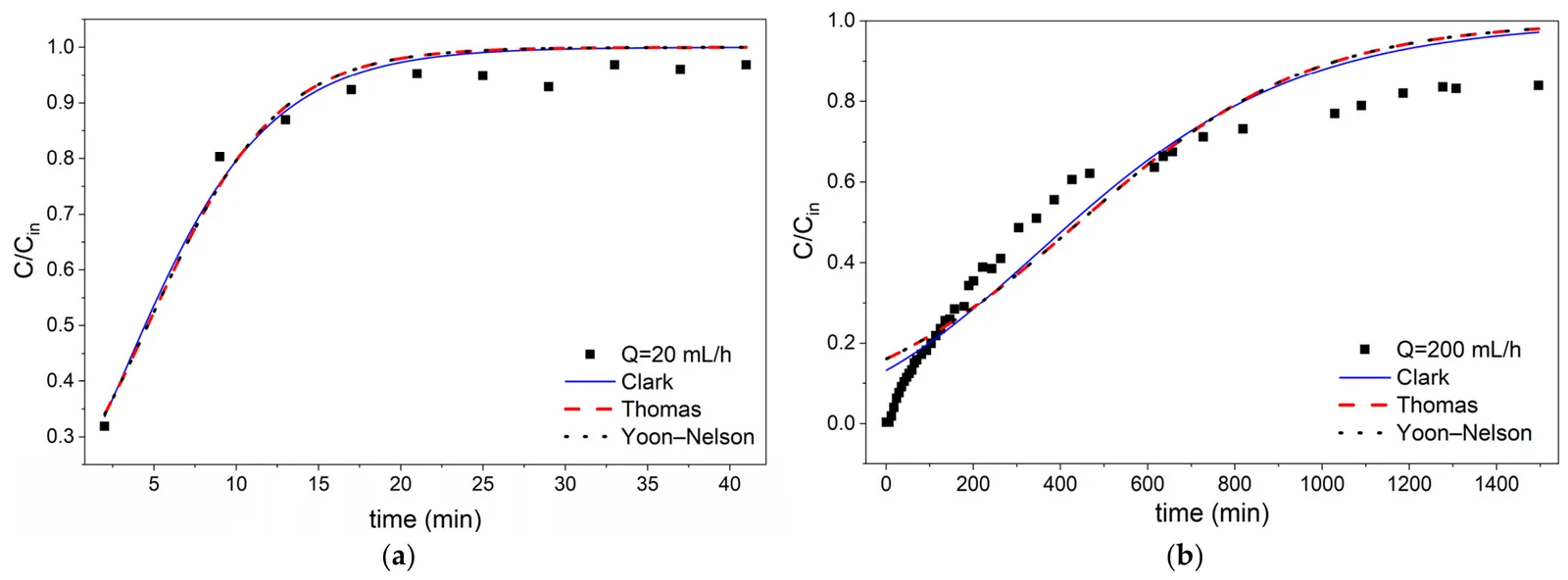
Breakthrough curves, i.e., dimensionless TPC concentration as a function of time for phenolic adsorption of the LfWL fitted using Clark, Thomas and Yoon–Nelson kinetic models for the (**a**) SFB-20 and (**b**) LFB-200 columns.

Following the batch assessment, continuous flow adsorption was evaluated using a small fixed-bed configuration operated at a flow rate of 20 mL/h (SFB-20) and a larger fixed-bed configuration operated at 200 mL/h (LFB-200). The breakthrough parameters for both configurations are summarized in Table 5. Both column configurations were operated in upflow mode to eliminate channeling phenomena and ensure a uniform flow distribution through the resin bed. In order to describe the breakthrough behavior, the experimental data were fitted to three fixed-bed kinetic models: the Thomas model, the Clark model and the Yoon–Nelson model (Figure 4). In SFB-20, the dimensionless TPC concentration (C/C_in_) increased sharply and approached unity within approximately 50 min. In contrast, LFB-200 exhibited a more gradual breakthrough profile, with the adsorption experiment extending beyond 1400 min. This prolonged process enhances steady-state interactions between the resin active sites and the target phenols. This happens because when a column saturates slowly and gradually, phenols have more time to travel deep into the inner pores of the resin, allowing it to use its full capacity [47].

The two fixed-bed configurations exhibited markedly different breakthrough profiles despite being operated at the same superficial velocity of 0.47 cm/min. Maintaining superficial velocity ensured that the flow rate increased approximately in proportion to the column cross-sectional area. Nevertheless, constant superficial velocity alone does not establish complete hydrodynamic similarity because the two configurations also differ in bed height, EBCT, aspect ratio and inlet TPC concentration.

The experimental adsorption capacity increased from 2.75 mg SAE/g for SFB-20 to 154.70 mg SAE/g for LFB-200, while the adsorption efficiency increased from 14.10% to 36.59%. In addition to the larger bed volume ($V_{b})$ (25-fold increase from 3.05 mL to 77.75 mL) and resin loading of LFB-200, this column was operated with an undiluted LfWL, resulting in a higher inlet TPC, and exhibited a greater bed height and EBCT (23.35 min compared with 9.24 min for SFB-20). The increased EBCT may have favored the diffusion of the complex phenolic molecules into the pores of the Amberlite XAD-16 resin [46]. Moreover, the LfWL feed was diluted for SFB-20 because of its substantially smaller resin mass and bed volume, thereby reducing the phenolic loading applied to the column and allowing its breakthrough behavior to be experimentally monitored. Therefore, the improved adsorption performance of LFB-200 likely reflects the combined influence of feed concentration, resin loading, bed dimensions, and residence time, highlighting the importance of appropriate column design and operating conditions for the continuous flow treatment of the LfWL.

Among the evaluated breakthrough kinetic models, the Clark model provided the highest R^2^ and lowest RMSE values for both configurations (Table 6), indicating that it provided the best statistical description of the experimental breakthrough curves among the models tested. The Clark model combines a mass transfer approach with a Freundlich-type equilibrium relationship [48]. Under continuous flow conditions, the shape of the breakthrough curve may be influenced by several transport phenomena, including external film mass transfer, intraparticle diffusion, and axial dispersion, as well as by operating parameters such as inlet concentration, bed height and flow conditions [49]. For LFB-200, the models predicted a non-zero C/C_in_ at the beginning of the run and slightly underestimated the data between 250 and 500 min, while they overestimated them after 800 min. The experimental breakthrough profile was therefore more asymmetric and exhibited a longer tail than the fitted curves. During fixed-bed adsorption of ferulic acid onto XAD-16, Dávila-Guzmán et al. applied a mass transfer model incorporating both axial dispersion and intraparticle diffusion, which provided a better description of the experimental breakthrough data than the Thomas model. In that system, intraparticle diffusion was identified as the rate-limiting resistance [50]. In contrast, the Thomas model assumes negligible axial dispersion and negligible internal and external mass transfer resistances. Taking into account these limitations, the better statistical performance of the Clark model in our experiments may reflect the mass transfer limitations, including intraparticle diffusion limitations. The observed asymmetry and tailing may also be related to the multicomponent nature of the TPC, which comprises phenolic compounds with different adsorption affinities and transport characteristics [49,51].

The present fixed-bed experiments demonstrate the feasibility of recovering phenolic compounds directly from the LfWL under continuous flow conditions and provide an initial indication that the process can be applied to a larger column configuration operating with undiluted feed and increased throughput. Nevertheless, further investigation under controlled scale-up conditions is required to assess long-term operational stability and resin regeneration.

#### 2.2.1. Desorption Experiments Results

**Figure 5:**
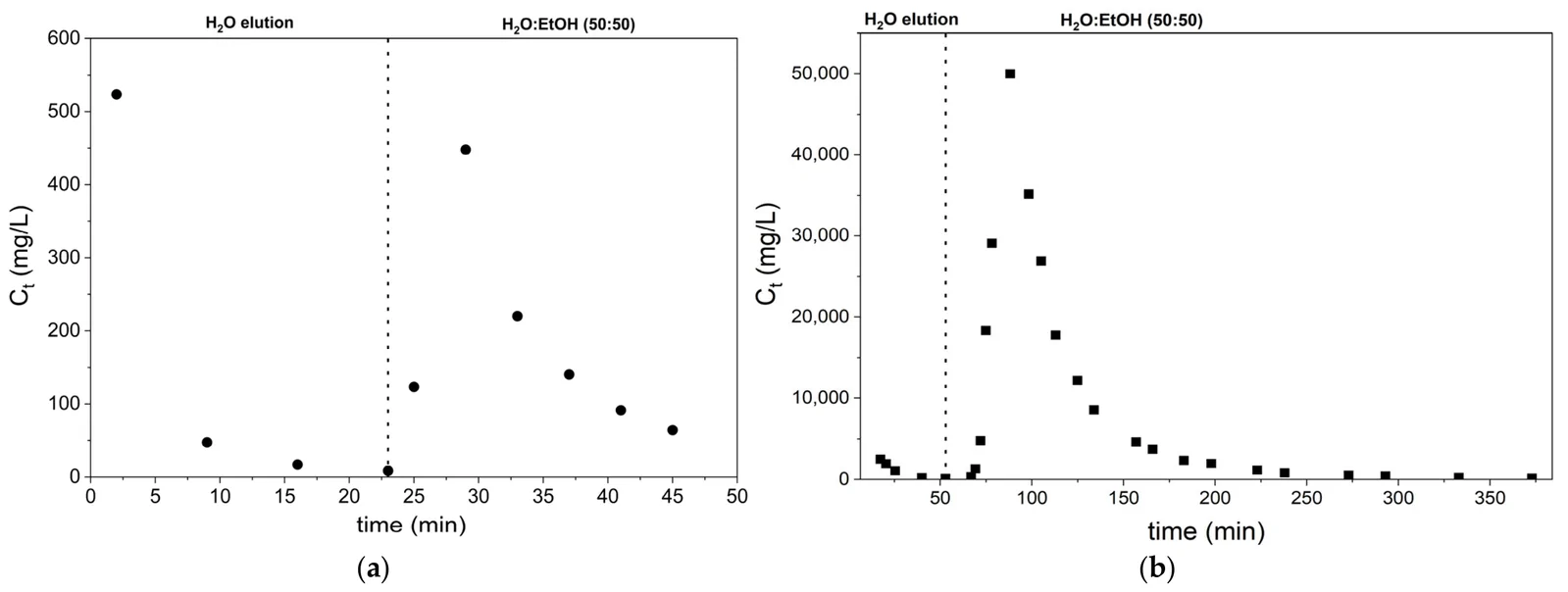
(**a**) Desorption curve at 20 mL/h for the SFB-20 column. (**b**) Desorption curve at 200 mL/h for the LFB-200 column.

Following continuous adsorption, a sequential desorption procedure was applied to recover the phenolic compounds retained on the XAD-16 resin. The adsorption experiments in SFB-20 and LFB-200 were followed by a two-stage desorption process. Initially, water was injected at 20 and 200 mL/h flow rates, respectively, because a water washing step removes readily elutable polar matrix constituents, including carbohydrates, from the resin surface [52], prior to phenolic desorption. In the second stage, a 50% *v*/*v* water–ethanol mixture was injected, maintaining the same flow rates as the first desorption stage. This specific hydroethanolic mixture was proven to be very efficient for the selective desorption of phenolic compounds in previous studies involving desorption of phenolic compounds from grape marc [53]. The transition between these two sequential desorption stages is clearly distinguished by the vertical dashed lines illustrated in Figure 5.

The desorption results (Table 7) showed that during desorption with water as the eluent, only a small amount of phenolic compounds was desorbed. In contrast, desorption using the water–ethanol mixture desorbed the majority of adsorbed phenols. Desorption efficiency (%Des) with water for SFB-20 (24.48%) was higher than for LFB-200 (2.45%) upon injecting 7.67 mL and 176.67 mL of water, respectively. On the other hand, during the water–ethanol mixture injections (SFB-20 and LFB-200), most phenolic compounds were desorbed (64.48% and 77.09%) within approximately 25 and 150 min, respectively. These findings align with the hydrophobic nature of phenolic compounds and agree with observations reported in the literature [18,54]. Although %Ads and %Des are useful parameters for evaluating the adsorption and desorption performance of XAD-16 in these column configurations, the overall adsorption–desorption recovery (*η*_A–D_) was calculated to assess the overall performance of the column process under the investigated operating conditions. In particular, SFB-20 and LFB-200 exhibited an *η*_A–D_ of 12.56% and 29.10%, respectively.

#### 2.2.2. Qualitative Analysis of LfWL Desorption Samples Using HRMS Analysis

High-resolution mass spectrometry analysis was employed to investigate the phenolic composition of the LfWL samples collected during the desorption stages. The collected samples included water elution only (17.5 min) and 50% *v*/*v* water–ethanol mixture elution (75.0, 88.3, 125.0 min) on the LFB-200 column. Based on accurate precursor masses and direct MS/MS spectral library matching (Figure S2), twenty phenolic compounds were selected as the most relevant putatively annotated compounds (Table S2). These included phenolic acids, flaval-3-ols and procyanidins, flavonol/flavanonol derivatives, and stilbenes. Direct spectral matches were obtained for compounds such as gallic acid, caffeic acid-related features, syringic acid, p-coumaric acid, taxifolin, piceid and resveratrol, whereas structurally ambiguous features were reported using more conservative annotations when the available MS/MS information did not permit clear discrimination of positional, stereochemical or glycosidic isomers. For example, the two features at *m*/*z* 289.0717 were reported as catechin/epicatechin isomers.

The quality of the spectral matches provided substantial support for the proposed annotations. Gallic acid, for example, was detected at *m*/*z* 169.01425 and RT 1.998 min and showed a direct library match score of 0.951 with nine matched MS/MS signals and essentially complete explained fragment intensity. Similarly, *p*-coumaric acid (*m*/*z* 163.04011, RT 8.404 min) exhibited a spectral similarity score of 0.974 with five matched fragment ions, whereas taxifolin (*m*/*z* 303.05103, RT 10.319 min) showed a score of 0.895 supported by 33 matched signals. These assignments should be regarded as putative annotations rather than confirmed compound identifications because authentic standards were not analyzed under identical chromatographic conditions [55].

**Figure 6:**
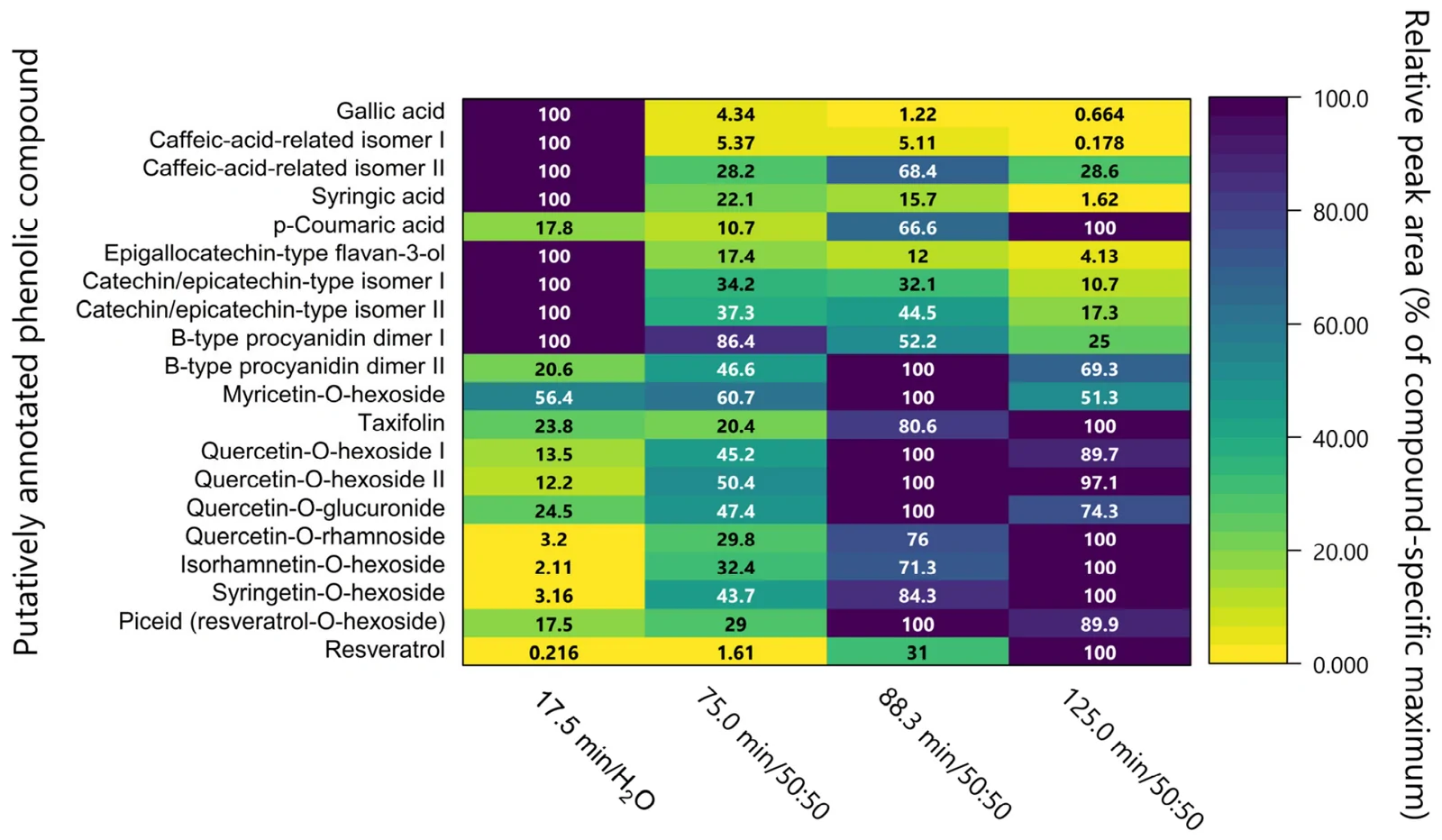
Relative distribution (heatmap) of putatively annotated phenolic compounds across XAD-16 desorption fractions obtained from the LFB-200 column. Peak areas were normalized independently for each compound to its maximum response across the four fractions (100%). The 17.5 min fraction corresponds to H_2_O elution, whereas the 75.0, 88.3, and 125.0 min fractions correspond to H_2_O:EtOH (50:50, *v*/*v*) elution.

A pronounced dependence of the phenolic profile on the desorption experiment onto XAD-16 was observed. To visualize these changes independently, the peak area profile of each compound was normalized to each maximum signal across the four collected fractions. The resulting heatmap (Figure 6) described the relative distribution of each individual compound throughout the desorption and should not be interpreted as an absolute comparison of concentrations between different compounds. The water elution sample at 17.5 min showed the maximum relative response for several phenolic acids and flavan-3-ol-related features, including gallic acid, both caffeic acid isomers, syringic acid, the epigallocatechin-type flavan-3-ol, both catechin/epicatechin type isomers and the first B-type procyanidin dimer. In contrast, several compounds exhibited progressively stronger responses during the water/ethanol elution stage. *p*-Coumaric acid reached its maximum relative response at 125.0 min, whereas the second B-type procyanidin dimer and myricetin-O-hexoside peaked at 88.3 min. Most flavonol derivatives, including taxifolin, quercetin-O-glycosides, isorhamnetin-O-hexoside and syringetin-O-hexoside, were predominantly associated with the 88.3 and 125.0 min fractions. The stilbenes showed a similarly delayed profile, with piceid reaching its maximum at 88.3 min and resveratrol at 125.0 min. Resveratrol, for example, increased from approximately 0.22% of its compound-specific maximum response at 17.5 min to 100% at 125.0 min. This distribution indicated that the water elution removed part of the more readily desorbed phenolic fractions, while the majority of the later-eluting flavonoid and stilbene features remained associated with the resin. Compound-dependent desorption from XAD-16 has also been reported previously [56], while a 50% water–ethanol mixture has been shown to be effective for the desorption of phenolic compounds from resin.

**Figure 7:**
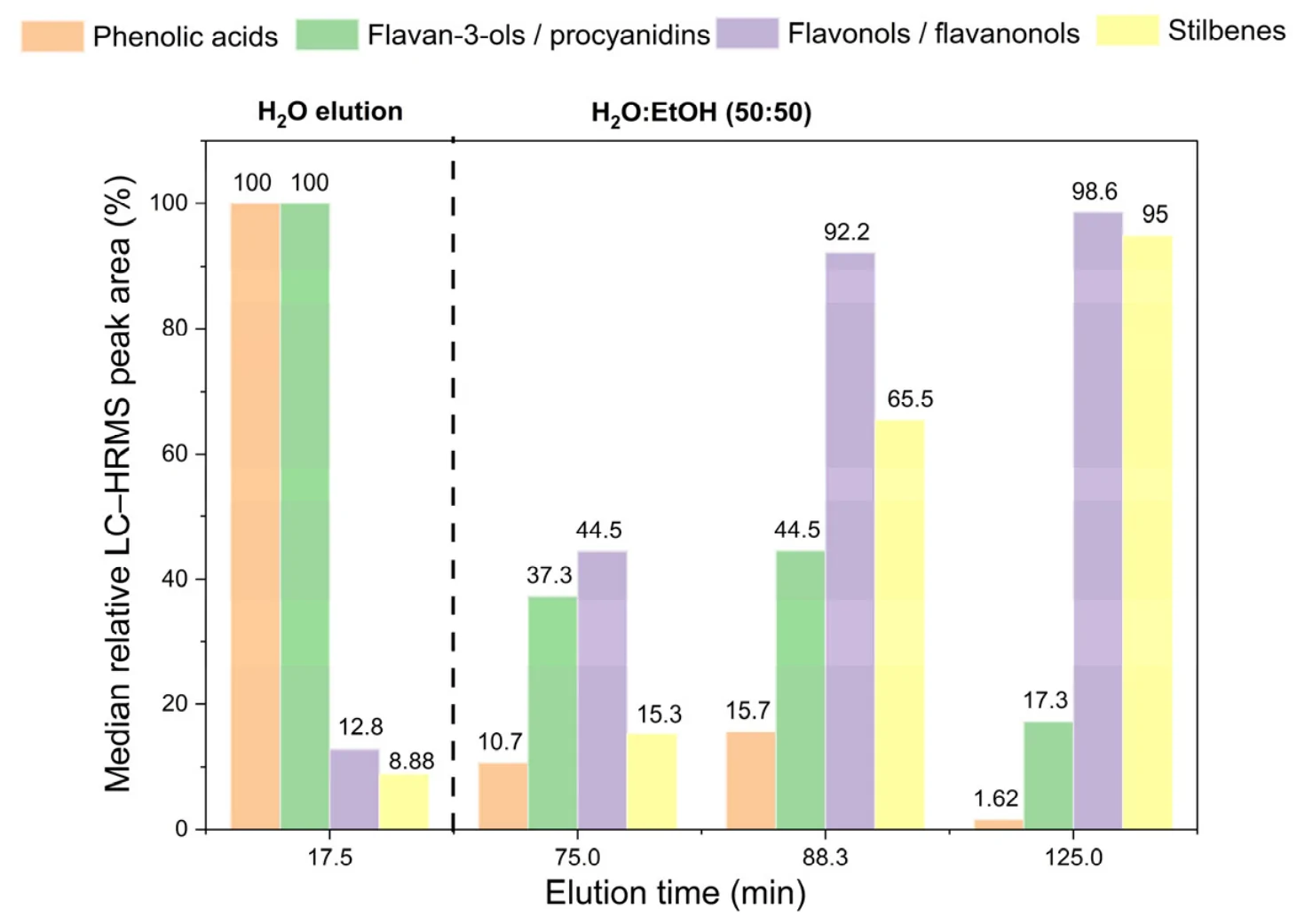
Class-level desorption profiles of putatively annotated phenolic compounds. Bars represent the median relative LC-HRMS peak area of compounds within each structural class after normalization of each compound to its maximum response across the four collected fractions (100%). Phenolic acids (*n* = 5); flavan-3-ols/procyanidins (*n* = 5); flavonols/flavanonols (*n* = 8); stilbenes (*n* = 2).

**Figure 8:**
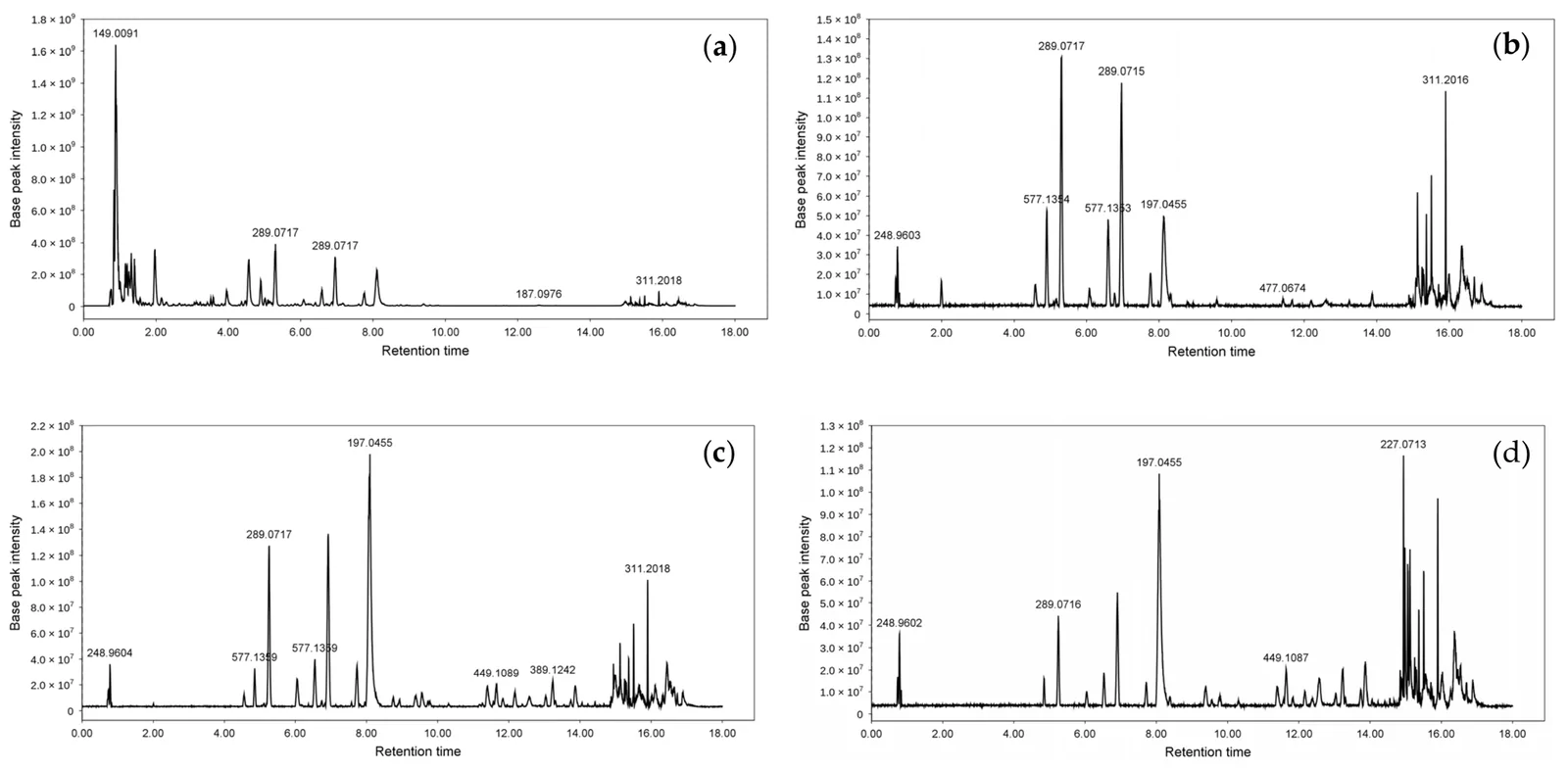
Base peak chromatograms of the four LFB-200 eluate fractions analyzed by LC-HRMS in negative ionization mode. The 17.5 min fraction corresponds to water elution (**a**), whereas the 75.0 (**b**), 88.3 (**c**), and 125.0 (**d**) min fractions correspond to H_2_O:EtOH (50:50, *v*/*v*) elution. Selected dominant ions are labeled by their experimental *m*/*z* values.

This trend was also evident at the structural class level (Figure 7). Phenolic acids and flaval-3-ols/procyanidins displayed the highest median relative responses at 17.5 min, whereas flavonols/flavanonols and stilbenes shifted markedly toward the later hydroethanolic fractions. Although these values represent compound-wise normalized LC-HRMS responses rather than absolute concentrations, they clearly demonstrate distinct class-dependent desorption patterns. Consequently, the HRMS profiles (Figure 8) suggest that XAD-16 enabled a temporally differentiated release of phenolic compounds during desorption. From a process perspective, the water-eluted fraction was characterized mainly by phenolic acids and flavan-3-ol-related compounds, whereas prolonged desorption favored flavonoid derivatives and stilbenes, indicating that fraction collection could be exploited to obtain eluates with distinct phenolic profiles.

## 3. Materials and Methods

### 3.1. Wine Lees Preparation and Characteristics

The red wine lees used for the batch and column experiments were collected from a Greek wine estate with vineyard holdings in Naoussa. The lees were first centrifuged and then vacuum-filtered to remove the heavy solid pulp fraction. With this treatment, the liquid fraction of red wine lees (LfWL) was collected for further use in the experiments. The total phenolic content (TPC) of the LfWL was found equal to 4709.8 ± 154.6 mg Syringic acid equivalents (SAE)/L with a pH value of 3.6 ± 0.2.

### 3.2. Adsorbent Material

The commercial macroporous polymeric adsorbent used in this study was AmberLite^TM^ XAD-16 (Sigma Aldrich, Burlington, MA, USA). According to the manufacturer’s specifications, it is a non-ionic, hydrophobic resin that consists of a divinylbenzene-crosslinked copolymer in the form of white translucent beads, exhibiting a high specific surface area (800 m^2^/g) and a porosity value of 0.55. Due to its high initial moisture-holding capacity (62–72%), the wet resin was dried in an oven at 50 °C and then stored in a desiccator.

### 3.3. Batch Kinetic Experiments

Batch kinetic adsorption experiments were conducted in 600 mL glass beakers, each containing a constant LfWL volume of 200 mL. Appropriate amounts of dried XAD-16 resin were added to the beakers to achieve adsorbent dosages of 20, 60, 80, 100, and 120 g/L. Agitation was maintained using a jar tester VELP Scientifica JLT6 (VELP Scientifica, Usmate Velate, Italy) at a constant speed of 150 rpm. To monitor adsorption kinetics, an aliquot of 2 mL was obtained at various time intervals. For the dosages of 60, 80, 100 and 120 g/L, the sampling was performed at 0, 5, 15, 30, 45, 60, 120, 150, 180, 210, and 240 min, while for the dosage of 20 g/L, sampling was extended to 300, 360, and 420 min in order to reach adsorption equilibrium. During each sampling, the jar tester was briefly paused to allow the resin beads to settle down. The equilibrium TPC values of the LfWL obtained from the kinetic experiments were used for isotherm fitting, where the initial TPC concentration of the LfWL was maintained constant while the XAD-16 dosage was varied as described above. This design was selected because resin dosage optimization under direct treatment of the LfWL matrix was a primary objective of the batch experiments and may better reflect a practical application in which feed composition remains fixed while adsorbent loading is adjusted according to the required phenolic removal. The effect of pH was examined as well. The raw effluent was pH 3.6, and it was adjusted to four distinct target values of 2.0, 5.5, 7.5, and 10.0 by adding drops of H_2_SO_4_ 1M to achieve acidic conditions and NaOH 2.5M for alkaline values, which were measured using an HI2002-02 edge^®^ pH/ORP meter (Hanna Instruments, Limena, Italy). A fixed resin dosage of 80 g/L was used in all pH experiments. Each experiment was performed in duplicate, and the results were presented as the mean ± standard deviation (SD). Under the same operating conditions, two samples were taken from two independent experiments to calculate each mean value. All collected samples were subsequently quantified for TPC using the Folin–Ciocalteu (FC) method, with a syringic acid calibration curve (TPC mg SAE/L). The specific measurement was determined with a UV-VIS spectrophotometer Hack Lange DR 5000 (Hack, Loveland, CO, USA) at 760 nm. Although the FC assay is widely used for TPC determination, discrepancies between FC-based TPC values and chromatographic quantification may exist due to the presence of other reducing substances in the wine lees matrix, such as proteins, carbohydrates, and aromatic amino acids [57].

Using the TPC of samples in each time interval, the adsorption yield (Adsorption yield (%)), adsorption capacity ($q_{t}$, mg SAE/g) and the equilibrium adsorption capacity ($q_{e}$, mg SAE/g) were calculated according to the following equations [48]:

(1) $$Adsorption\;yield\;(\%)=\frac{C_{0}-C_{e}}{C_{0}}\cdot 100\%$$

(2) $$q_{t}=\frac{\left(C_{0}-C_{t}\right)\cdot V}{m}$$

(3) $$q_{e}=\frac{{(C}_{0}-C_{e})\cdot V}{m}$$

where $C_{0}$ and $C_{e}$ are the initial and equilibrium TPC values of the LfWL $\left(\frac{mg}{L}\right),$ respectively; $C_{t}$ is the TPC value of the LfWL in each time interval of the adsorption (mg/L); m is the mass of the resin $\left(g\right)$ and V is the volume of the LfWL (L).

#### 3.3.1. Adsorption Kinetic Models

To describe the adsorption mechanisms, the batch experimental data were modeled using the linear and non-linear pseudo-first-order (PFO) and pseudo-second-order (PSO) kinetic equations. The PFO model, also known as the Lagergren equation, describes solid–liquid adsorption systems where the rate of adsorption is governed by the sorption capacity of the adsorbent [58]. This model assumes that organic molecules establish a reversible equilibrium between the liquid and solid phases [59]. On the other hand, the PSO kinetic model assumes that the adsorption rate is proportional to the number of available active sites on the adsorbent’s surface [58].

The non-linear and linear forms of PFO are described by the following equations:

(4) $$q_{t}=q_{e}\left(1-e^{-k_{1}t}\right)$$

(5) $$\ln\left(\frac{q_{e}-q_{t}}{q_{e}}\right)=-k_{1}t$$

where $q_{e}$ and $q_{t}$ are the amount of adsorbed phenolic compounds (mg SAE/g) at equilibrium and at time t (min) and $k_{1}$ is the pseudo-first-order kinetic constant (1/min) [23,58].

The non-linear and linear forms of PSO are expressed as follows:

(6) $$q_{t}=\frac{k_{2}tq_{e}^{2}}{1+k_{2}{tq}_{e}}$$

(7) $$\frac{t}{q_{t}}=\frac{1}{k_{2}q_{e}^{2}}+\frac{t}{q_{e}}$$

where $k_{2}$ is the pseudo-second-order kinetic constant (g/(min∙mg) [23,58].

#### 3.3.2. Adsorption Isotherm Models

To describe how the phenolic compounds from the LfWL are adsorbed onto the resin and evaluate their interactions, two models were applied, i.e., the non-linear and linear Langmuir models, which are expressed by Equations (8) and (9), and the non-linear and linear Freundlich models (Equations (10) and (11)).

(8) $$q_{e}=\frac{Q_{m}K_{L}C_{e}}{1+K_{L}C_{e}}$$

(9) $$\frac{1}{q_{e}}=\frac{1}{Q_{m}K_{L}}\cdot \frac{1}{C_{e}}+\frac{1}{Q_{m}}$$

(10) qe=KFCe1n

(11) $${log(q}_{e})=log(K_{F})+\frac{1}{n}log(C_{e})$$

where $Q_{m}$ is the maximum amount of phenol the resin can adsorb per gram of resin (mg/g), n is the Freundlich constant that indicates if the process is favorable (1/n < 1), and $K_{L}$ and $K_{F}$ are fitting constants unique to each model (in L/mg, and $\left(mg/g\right){\left(L/mg\right)}^{1/n}$, respectively) [26].

The Langmuir model works under several simplifying assumptions: the resin surface is uniform, every adsorption site behaves the same way, adsorbed molecules do not affect each other, there is only one adsorption mechanism, and phenols form a single layer on the surface. However, these assumptions are not absolutely realistic since different types of sites exist on the surface and molecules may interact between each other [59]. The Freundlich model, on the other hand, is an empirical model better suited for real, non-ideal systems where the surface is heterogeneous. It doesn’t require the formation of a single layer, and it tends to fit experimental data well within a limited concentration range [59].

#### 3.3.3. Adsorption Thermodynamics

To better understand the nature of the interaction between LfWL phenolic compounds and the adsorbent, key thermodynamic parameters were calculated, specifically standard Gibbs free energy ($\Delta G^{0}$), enthalpy change ($\Delta H^{0}$), and entropy change ($\Delta S^{0}$). These parameters provide insight into whether the adsorption process occurs spontaneously and reveal the energetic characteristics of the interaction between the adsorbate and the adsorbent [23].

The apparent dimensionless distribution constant ($K_{d})$ was calculated according to:

(12) $$K_{d}=\frac{C_{Ad}}{C_{e}}$$

where $C_{Ad}\;(mg\;SAE/L)$ is the concentration of the phenolic compounds adsorbed onto the resin, calculated as $(C_{Ad}=C_{0}-C_{e}$), where $C_{0}\;(mg\;SAE/L)$ and $C_{e}\;(mg\;SAE/L)$ represent the initial and equilibrium TPC concentrations, respectively, at each temperature. Both terms were expressed in mg SAE/L and referred to the same standard state, a hypothetical ideal solution of unit molarity (C° = 1 mol/L). Using K_d_ as an approximation of the thermodynamic equilibrium constant additionally assumes that activity coefficients are close to unity [60].

The standard Gibbs free energy was calculated according to:

(13) $$\Delta G^{0}=-RT\;lnK_{d}$$

where R is the gas constant (8.314 J/mol·K) and T(K) is the temperature.

The standard enthalpy and entropy change were calculated using the Van’t Hoff equation:

(14) $$lnK_{d}=-\frac{\Delta H^{0}}{RT}+\frac{{\Delta S}^{0}}{R}$$

The values of $\Delta H^{0}$ and ${\Delta S}^{0}$ were calculated from the slope and intercept of the plot of ln $K_{d}$ versus 1/T (1/K) [23,61]. Because activity coefficients in a real wine lees matrix may deviate from unity and $K_{d}$ is evaluated at a fixed adsorbent dosage rather than extrapolated to infinite dilution, the resulting ΔG°, ΔH°, and ΔS° values represent apparent thermodynamic parameters.

### 3.4. Fixed-Bed Experiments

Continuous flow adsorption studies were performed to evaluate the dynamic behavior of phenolic compound adsorption under fixed-bed conditions. Two distinct configurations at different scales were utilized: a Small Fixed Bed with a volumetric flow rate of 20 mL/h (SFB-20) and a Large Fixed Bed with a volumetric flow rate of 200 mL/h (LFB-200). SFB-20 had an internal diameter of 0.95 cm, a bed height of 4.3 cm, and a bed volume of approximately 3.05 mL, packed with 0.8276 g dry resin, while LFB-200 had 3 cm, 11 cm, 77.75 mL, 52 g, respectively. The LfWL solution was diluted with deionized water at a 1:4 *v*/*v* ratio prior to injection into SFB-20, while the LfWL solution was fed undiluted into LFB-200. Injection was done with a peristaltic pump. All dynamic experiments were conducted under ambient conditions. Samples were collected at various time intervals from the top of the columns (upflow). From the column specifications and the data obtained from the experiments, the following parameters were determined:

(15) $$EBCT=\frac{V_{b}}{Q}\;$$

where EBCT is the empty bed contact time (min), $V_{b}$ is the bed volume (mL) and Q is the volumetric flow rate (mL/min), and

(16) $$u_{0}=\frac{4Q}{\pi D_{in}^{2}}$$

where $u_{0}$ is the superficial velocity (cm/min) and $D_{in}$ is the internal diameter of each column (cm).

The adsorption capacity ($q_{e,exp},mg\;SAE/g$) and the adsorption efficiency (%Ads) were determined based on Equations (17)–(20):

(17) $$q_{total}=\frac{Q}{1000}\int_{t=0}^{t=t_{total}}C_{ads}dt$$

(18) $$q_{e,exp}=\frac{q_{total}}{x}$$

(19) $$W_{total}=\frac{C_{0}Qt_{total}}{1000}$$

(20) $$\%Ads=\frac{q_{total}}{W_{total}}\cdot 100$$

where $C_{ads}$ are the adsorbed phenolic compounds (mg/L) (calculated as $C_{ads}=C_{0}-C_{t}$), Q is the volumetric flow rate (mL/min), $q_{total}$ is the amount of adsorbed phenolic compounds (mgSAE) calculated from the area under the $C_{ads}(t)$ curve plot in Origin software (v2026b) and the volumetric flow rate, $t_{total}$ is 90% of the time needed for adsorbent saturation, x the total dry mass of the resin inside the column (g) and $W_{total}$ is the total amount of phenolic compounds (mgSAE) introduced into the column [47].

#### 3.4.1. Breakthrough Kinetic Models

In order to understand the dynamic behavior of the column, mathematical breakthrough kinetic models, including the Thomas, Yoon–Nelson, and Clark models, were employed.

The Thomas model assumes that adsorption kinetics obey the Langmuir isotherm principles, the rate of adsorption is governed by chemical reaction effects rather than diffusion resistances, and the packed bed operates under ideal plug-flow with negligible axial dispersion. Consequently, this model is suitable for evaluating adsorption systems characterized by minimum external and internal mass transfer resistances [21,23,62].

The non-linear Thomas model is expressed by Equation (21):

(21) $$\frac{C_{t}}{C_{0}}=\frac{1}{1+\exp\left(k_{Th}C_{0}\left(\frac{q_{Th}x}{F_{v}C_{0}}-t\right)\right)}$$

where $C_{0}$ is the initial TPC (mg/L), $C_{t}$ is the effluent concentration (mg/L), $F_{v}$ the flow rate (mL/min), $k_{Th}$ is the Thomas adsorption rate constant (mL/(mg·min)), $q_{Th}$ is the adsorption capacity (mg/g) and x is the mass of the adsorbent (g).

The Yoon–Nelson model provides a simple description of fixed-bed adsorption analysis, as it eliminates the need for detailed information regarding column physical parameters or adsorbent–adsorbate interactions. It assumes that the rate of decrease in the probability of adsorption is proportional to the product of the probabilities of adsorption and breakthrough [21,23,62].

The non-linear Yoon–Nelson model is expressed by Equation (22):

(22) $$\frac{C_{t}}{C_{0}}=\frac{\exp\left(k_{YN}\left(t-\delta \right)\right)}{1+\exp\left(k_{YN}\left(t-\delta \right)\right)}$$

where $k_{YN}$ is the Yoon–Nelson constant (1/min) and δ the time required for 50% saturation (min).

The Clark model is used to predict the fixed-bed breakthrough curve based on the principles of adsorption equilibrium and mass transfer phenomena, specifically film diffusion. The assumption of this model is that the interactions between the adsorbent and the adsorbate are described by the Freundlich isotherm [48,62]. The non-linear Clark model is expressed by Equation (23):

(23) $$\frac{C_{t}}{C_{0}}=\frac{1}{{\left[1+\mathrm{Aexp}\left(-rt\right)\right]}^{\;\frac{1}{n-1}}}$$

where n is the Freundlich constant based on batch experiments and r(1/min) and A are kinetic- and breakthrough concentration-related constants, respectively.

#### 3.4.2. Desorption Experiments

Following the fixed-bed adsorption process, desorption experiments were conducted to evaluate recovery efficiency. The desorption process was carried out in two sequential stages using water as the first stage and then a water/ethanol 50% *v*/*v* mixture, while effluent samples were collected to calculate the mass of the phenolic compounds desorbed. The desorbed amount of phenolic substances ($m_{des})$, the desorption efficiency (%Des), and the overall adsorption–desorption recovery ($\eta_{A-D})$ were calculated using the following equations, respectively:

(24) $$m_{des}=Q\int_{0}^{t}C_{out}dt$$

(25) $$\%Des=\frac{m_{des}}{q_{total}}\cdot 100\;$$

(26) $$\eta_{A-D}=\frac{m_{des}}{W_{total}}\cdot 100$$

where $C_{out}$ is the TPC of the desorbed phenolic compounds in the outlet of the column $\left(mgL^{-1}\right)$, $m_{des}$ is the amount of desorbed phenolic substances (mg SAE) calculated from the area under the $C_{out}(t)$ curve plot in Origin software and the volumetric flow rate, $q_{total}$ is the amount of adsorbed phenolic compounds (mg SAE) calculated using Equation (17), Q is the volumetric flow rate (mL/min), t is the time needed for each desorption step (min) and $\eta_{A-D}$ represents the fraction of the total TPC mass fed to the column that was recovered during desorption.

### 3.5. HRMS Analysis

The recovered phenolic fractions obtained from the large fixed-bed adsorption–desorption process (LFB-200) were characterized by LC-HRMS/MS to provide compound-level information on the recovered phenolic profile. LFB-200 desorption fractions were chosen because they had higher TPC values than SFB-20 desorption fractions. Chromatographic separation was performed using a Thermo Scientific Vanquish system (Thermo Fisher Scientific, Waltham, MA, USA) equipped with a VF-P10-A binary pump, a VF-A10-A autosampler, and a Kinetex C18 column (150 × 2.1 mm, 2.6 μm, 100 Å), (Phenomenex, Torrance, CA, USA). The LC system was coupled through an H-ESI II source to an Orbitrap Exploris 120 mass spectrometer (Thermo Fisher Scientific, Waltham, MA, USA)). Data were acquired in negative electrospray ionization mode using data-dependent acquisition (DDA) with higher-energy collisional dissociation (HDC). Instrument control and data acquisition were performed using Xcalibur 4.0.7.

#### HRMS Data Processing

Raw LC-HRMS/MS data files (.raw format) were converted to open-source .mzML centroid format using the ProteoWizard MSConrvert tool (v3.0) (ProteoWizard, Palo Alto, CA, USA). Data processing was performed using the open-source software MZmine (v4.10.6) according to established MZmine LC-MS processing workflows, with settings selected according to the characteristics of the present Orbitrap LC-HRMS/MS dataset. The same software was previously employed for HRMS/MS annotation in non-targeted polyphenol analysis [63].

Mass detection on raw scans was conducted in centroid mode with an MS1 noise factor of 5.0 and an MS2 noise factor of 2.5. Chromatographic feature extraction was carried out using the Automated Data Analysis Pipeline (ADAP) Chromatographic Builder of the MZmine software within the RT range of 0.30–18.01 min. A minimum of four consecutive scans was set, and a minimum consecutive scan intensity of $2.0\times 10^{3}$, a minimum feature height of $1.0\times 10^{4}$, and an *m*/*z* tolerance of 5 ppm were applied. Peak resolution was performed using the local minimum search algorithm, applying a chromatographic threshold of 90%, a minimum search range of 0.05 min, a minimum absolute height of $1.0\times 10^{4}$, a minimum peak-top-to-edge ratio of 1.8, a maximum peak duration of 1.5 min, and at least four data points per feature. Isotropic features were grouped using the ^13^C Isotope Filter module (*m*/*z* tolerance: 3 ppm, RT tolerance: 0.08 min, maximum charge: 2). Feature alignment across all analyzed sample runs was performed using the Join Aligner module with an *m*/*z* tolerance of 5 ppm and an RT tolerance of 0.30 min. Gap filling was performed using the multithreaded finder with an intensity tolerance of 20%, an *m*/*z* tolerance of 5 ppm, an RT tolerance of 0.30 min, and a minimum of two data points.

Annotation and putative identification of phenolic compounds and organic acids were performed based on criteria established by the Metabolomics Standards Initiative [64] (MS1, Level 2, annotation). High-resolution precursor ions were matched in negative ionization mode ([M-H]^−^) with an error tolerance of 20 ppm, combined with MS/MS spectral matching against public and curated natural product spectral libraries (MassBank in NIST-compatible MSP format, MoNA LC-MS/MS Negative Mode, and the MSMS-Public-experimentspectra-neg-VS19 negative-mode spectral library). A minimum spectral cosine similarity score of 0.70 and at least 4 matching diagnostic MS/MS fragments were required for compound assignment [64].

### 3.6. Determination of Error Functions and Coefficients

The parameters obtained from the linear and non-linear models (kinetics, isotherms) were substituted into the corresponding original model equations to calculate the predicted adsorption capacities. To evaluate and compare all fits on the same basis, as well as to determine the uncertainty and error distribution, the coefficient of determination $\left(R^{2}\right)$ and root mean square error (RMSE) of the measurements were calculated using the experimental and predicted adsorption capacities. The mathematical definitions for these statistical indicators are expressed by Equations (27) and (28).

(27) $$R^{2}=\frac{\sum_{i=1}^{p}{\left(q_{e,theor}^{i}-{\bar{q}}_{e,means}^{i}\right)}^{2}}{\sum_{i=1}^{p}{\left((q_{e,theor}^{i}-{\bar{q}}_{e,means}^{i})+(q_{e,theor}^{i}-q_{e,means}^{i}\right)}^{2})}$$

(28) $$RMSE=\sqrt{\frac{1}{n-p}\sum_{i=1}^{n}{\left(q_{e,theor}^{i}-q_{e,means}^{i}\right)}^{2}}$$

where i is the experimental run, $q_{e,theor}$ is the predicted calculated equilibrium values from the model (mg/g), $q_{e,means}$ is the experimental equilibrium data (mg/g), n is the number of experimental data points (at least 5) and p is the number of fitted parameters [48].

## 4. Conclusions

This study demonstrated the potential of Amberlite XAD-16 resin for the direct recovery of phenolic compounds from the liquid fraction of red wine lees, avoiding the need for an additional solid–liquid extraction step. Batch experiments showed that adsorption efficiency was strongly influenced by resin dosage, while the natural pH of the wine lees (pH 3.6) provided favorable adsorption conditions. The adsorption kinetics were better described by the pseudo-second-order model, whereas the non-linear Langmuir model provided the best representation of the equilibrium data, with a model-estimated maximum adsorption capacity of 304.34 mg SAE/g. Thermodynamic analysis indicated that adsorption was thermodynamically favorable and exothermic under the investigated conditions.

The fixed-bed experiments further demonstrated the applicability of XAD-16 under continuous flow conditions, with the larger column providing a later breakthrough profile and allowing direct processing of an undiluted LfWL. Sequential desorption showed that the hydroethanolic phase accounted for the major fraction of recovered phenolics, while desorption yields were comparable between the two column configurations. The higher overall adsorption–desorption recovery achieved with LFB-200 (29.10% compared with 12.59% for SFB-20) further emphasizes the importance of column configuration and operating conditions in the continuous flow recovery of phenolic compounds from the LfWL. LC-HRMS/MS analysis enabled the putative annotation of 20 phenolic compounds and revealed distinct compound- and class-dependent elution profiles. Several phenolic acids and flaval-3-ol-related compounds exhibited earlier responses, whereas flavonol derivatives and stilbenes were predominantly associated with later hydroethanolic fractions.

In conclusion, the combined adsorption–desorption and LC-HRMS approach supports the direct valorization of the liquid fraction of red wine lees as a source of recoverable phenolic compounds. Despite the promising adsorption and desorption performance observed, further work is required to assess the robustness and scalability of the process. Resin regeneration, repeated adsorption–desorption cycles, long-term fixed-bed operation, and optimization of operating conditions should be investigated under controlled scale-up conditions. In addition, confirmation of the putatively annotated phenolic compounds using authentic standards would strengthen the analytical characterization of the recovered fractions.

## Figures and Tables

**Table 1:** Kinetic parameters of the linear model fittings for adsorption of phenolic compounds from LfWL onto XAD-16 resin.

XAD-16		PFO			PSO			
Resin Dosage (g/L)	q_e,exp_ (mg/g)	k_1_ (1/min)	R^2^	RMSE (mg/g)	q_e,cal_ (mg/g)	k_2_ (g/(mg·min))	R^2^	RMSE (mg/g)
20	123.49	0.00875	0.978	19.69	132.28	0.00019	0.997	3.49
60	64.17	0.01903	0.976	9.21	68.26	0.00084	0.999	2.28
80	51.45	0.02229	0.933	10.79	53.13	0.00233	0.999	2.00
100	42.76	0.02032	0.914	10.44	43.73	0.00364	0.999	0.54
120	34.11	0.02203	0.921	9.02	34.76	0.00565	0.999	0.30

**Table 2:** Kinetic parameters of the non-linear model fittings for adsorption of phenolic compounds from LfWL onto XAD-16 resin.

XAD-16		Non-Linear PFO				Non-Linear PSO			
Resin Dosage (g/L)	q_e,exp_ (mg/g)	q_e,cal_ (mg/g)	k_1_ (1/min)	R^2^	RMSE (mg/g)	q_e,cal_ (mg/g)	k_2_ (g/(mg·min))	R^2^	RMSE (mg/g)
20	123.49	115.31	0.022	0.981	6.00	130.85	0.00021	0.995	3.05
60	64.17	60.06	0.044	0.954	4.44	66.45	0.00099	0.990	2.10
80	51.45	48.54	0.099	0.943	3.78	51.90	0.00302	0.989	1.68
100	42.76	40.91	0.104	0.975	2.08	43.56	0.00383	0.998	0.51
120	34.11	32.66	0.131	0.980	1.53	34.60	0.00608	0.999	0.14

**Table 3:** Isotherm parameters of the Langmuir and Freundlich non-linear models for LfWL adsorption.

Model	Parameters	Value
Non-linear Langmuir	Q_m_ (mg/g)	304.34
	K_L_ (L/mg)	3.04 × 10^−4^
	R^2^	0.9941
	RMSE (mg/g)	3.151
Non-linear Freundlich	1/n	0.74
	K_F_ (mg/g) (L/mg)^1/n^	0.43
	R^2^	0.9923
	RMSE (mg/g)	3.587

**Table 4:** Thermodynamic parameters from the Van’t Hoff plot for LfWL adsorption.

T (K)	ΔG^0^ (kJ/mol)	ΔH^0^ (kJ/mol)	ΔS^0^ (J/(mol·K))	R^2^
293.15	−4.75	−47.78	−147.29	0.9634
303.15	−2.81			
313.15	−1.83			

**Table 5:** Breakthrough parameters for the two column configurations.

	SFB-20	LFB-200
Q (mL/min)	0.33	3.33
Bed volume (V_b_) (mL)	3.05	77.75
Dry resin mass (x) (g)	0.8276	51.00
EBCT (min)	9.24	23.35
u_0_ (cm/min)	0.47	0.47
TPCi (mg SAE/L)	1098	4407
q_total_ (mg SAE)	2.12	8044.48
q_e,exp_ (mg SAE/g)	2.56	154.70

**Table 6:** Fitted parameters for Thomas, Clark, and Yoon–Nelson kinetic models for both columns.

	Thomas				Clark				Yoon–Nelson			
	k_Th_ (mL/(mg∙min))	q_Th_ (mg/g)	R^2^	RMSE	A	r (1/min)	R^2^	RMSE	k_YN_ (1/min)	τ (min)	R^2^	RMSE
SFB-20	0.231	2.04	0.952	0.039	0.717	0.215	0.958	0.036	0.253	4.61	0.952	0.039
LFB-200	0.000846	125.33	0.894	0.090	1.031	0.0031	0.920	0.078	0.0039	443.65	0.894	0.090

**Table 7:** Mass balance parameters for column adsorption and desorption of phenolic compounds in the LfWL.

Adsorption Experiment		TPC Injected (mg)	TPC Adsorbed (mg)	%Ads	*η*_A–D_ (%)
SFB-20 (TPCi = 1098 mg/L)		15.01	2.12	14.10	12.56
LFB-200 (TPCi = 4407 mg/L)		21,983.6	8044.48	36.59	29.10
**Desorption Experiment**	**Solvent Used**	**Solvent Volume Injected (mL)**	**TPC** **Desorbed (mg)**	**%Des**	
SFB-20	Water	7.67	0.519	24.48	
	Water–EtOH mixture; 50% *v*/*v*	7.33	1.367	64.48	
LFB-200	Water	176.67	197.00	2.45	
	Water–EtOH mixture; 50% *v*/*v*	1066.7	6201.3	77.09	

## Data Availability

The data presented in this study are available within this article and the Supplementary Materials. Additional raw data supporting the findings of this study are available from the corresponding authors upon reasonable request.

## Supplementary Materials

The following supporting information can be downloaded at https://www.mdpi.com/article/10.3390/molecules31213929/s1, Figure S1: Non-linear regression of isotherm models for the adsorption of phenolic compounds from LfWL onto XAD-16 resin: (a) Langmuir; (b) Freundlich.; Figure S2: Mirror plots of the experimental and spectral libraries (MS2 match) for (a) Resveratrol; (b) Gallic acid; (c) Taxifolin.; Table S1: Isotherms parameters of Langmuir and Freundlich linear models for LfWL adsorption.; Table S2: Putative identification of phenolic compounds in XAD-16 eluates by LC-HRMS/MS and spectral-library matching.

## Abbreviations

The following abbreviations are used in this manuscript:

LfWL	Liquid fraction of red wine lees
TPC	Total phenolic content
GAE	Gallic acid equivalents
SAE	Syringic acid equivalents
LC-HRMS/MS	Liquid chromatography coupled to high-resolution tandem mass spectrometry
LFB-200	Large fixed-bed configuration operated at 200 mL h^−1^
SFB-20	Small fixed-bed configuration operated at 20 mL h^−1^
MSI	Metabolomics Standards Initiative
OIV	International Organisation of Vine and Wine
PFO	Pseudo-first-order
PSO	Pseudo-second-order
RMSE	Root mean square error
RT	Retention time
EBCT	Empty bed contact time
