# Supplementary material for: Red Wine Lees Valorization for Phenolic Compounds Recovery and Characterization Using Resin Adsorption
Source: Molecules. 2026 Nov 1;31(21):3929. doi: 10.3390/molecules31213929 (PMC13765274; doi:10.3390/molecules31213929)
Supplement: Supplementary file 1 [file molecules-31-03929-s001.zip › molecules-4652673-supplementary.pdf]

Supplementary Material

Red Wine Lees Valorization for Phenolic Compounds Recovery and Characterization Using Resin Adsorption

Antonis Vasilopoulos [1], Varvara Sygouni [1,*], Christina Vasiliki Lazaratou [1,*], Athanasia G. Tekerlekopoulou [2] and Dimitris V. Vayenas [1,3]

1 Department of Chemical Engineering, University of Patras, 26504 Patras, Greece; vasilopantonis@chemeng.upatras.gr (A.V.); dvagenas@upatras.gr (D.V.V.)
2 Department of Sustainable Agriculture, University of Patras, 30100 Agrinio, Greece; atekerle@upatras.gr
3 Foundation for Research and Technology—Hellas, Institute of Chemical Engineering Sciences, FORTH/ICE-HT, 26504 Patras, Greece
* Correspondence: sygouni@upatras.gr (V.S.); c.lazaratou@ac.upatras.gr (C.V.L.)

Table S1. Isotherms parameters of Langmuir and Freundlich linear models for LfWL adsorption.

Model	Parameters	Value
Linear Langmuir	Q_m (mg/g)	404.86
	K_L (L /mg)	2.15×10^{-4}
	R^2	0.9686
	RMSE (mg/ g)	5.771
Linear Freundlich	1/n	0.76
	K_F (mg/g) (L /mg)$^{1/n}$	0.35
	R^2	0.9783
	RMSE (mg/ g)	4.141

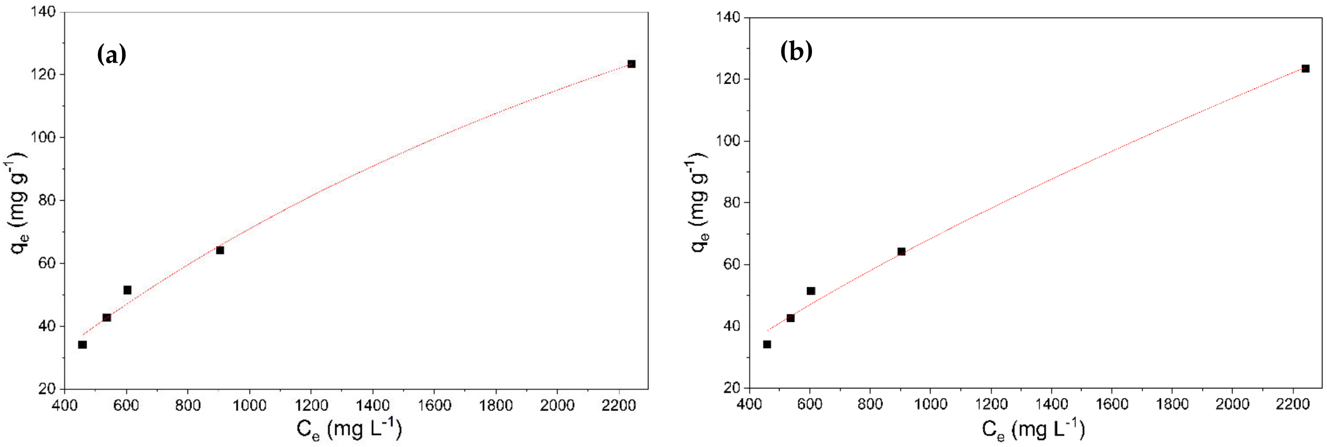

Figure S1. Non-linear regression of isotherm models for the adsorption of phenolic compounds from LfWL onto XAD-16 resin: (a) Langmuir; (b) Freundlich.

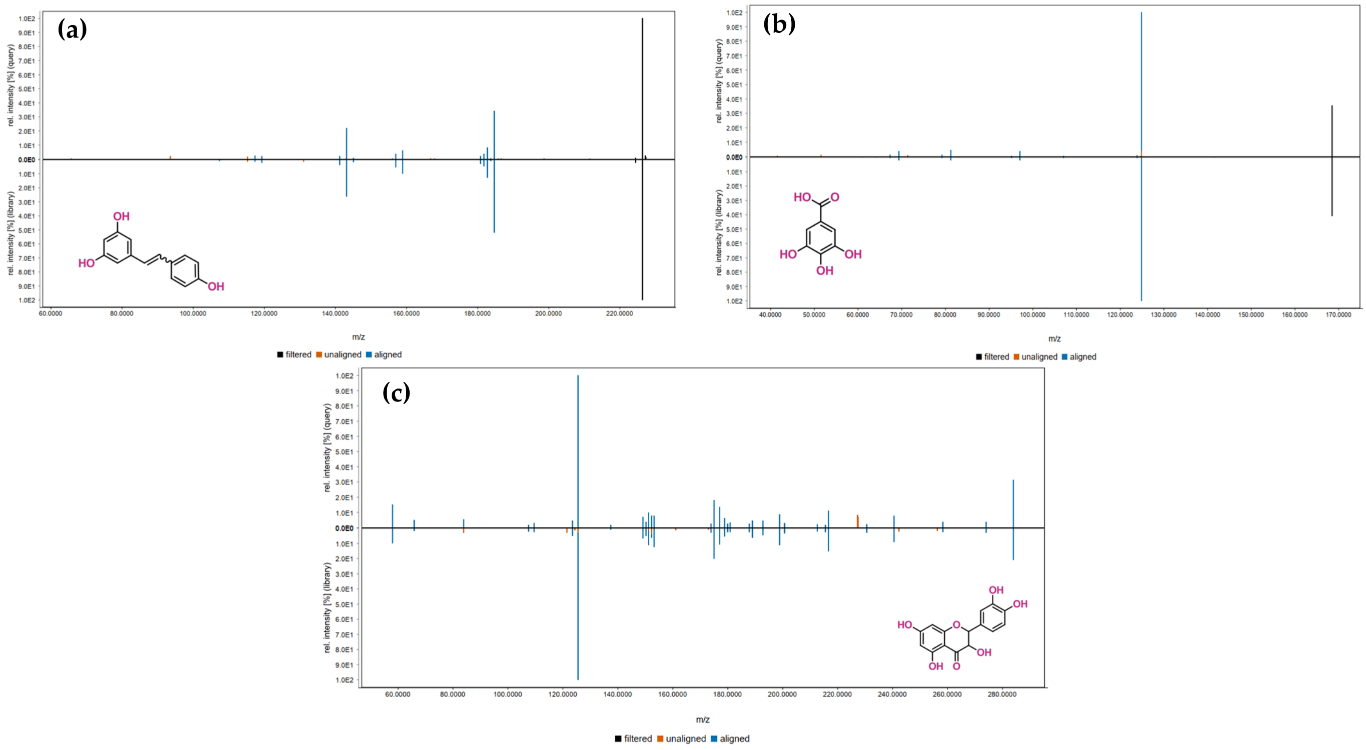

Figure S2. Mirror plots of the experimental and spectral libraries (MS2 match) for (a) Resveratrol; (b) Gallic acid; (c) Taxifolin.

Table S2. Putative identification of phenolic compounds in XAD-16 eluates by LC-HRMS/MS and spectral-library matching.

Putatively annotated compound	Molecular formula	Exp. [M−H]− (*m*/*z*)	RT (min)	Δm(ppm)	Spectral score	Matched MS/MS ions	Explained MS/MS intensity (%)	Confidence
Gallic acid	$C_7H_6O_5$	169.01426	1.9952	+0.08	0.951	9	99.27	MSI Level 2
Caffeic-acid-related isomer I	$C_9H_8O_4$	179.03501	3.9946	+0.15	0.962	4	99.56	MSI Level 2
Caffeic-acid-related isomer II	$C_9H_8O_4$	179.03500	6.0710	+0.10	0.961	5	99.69	MSI Level 2
Syringic acid	$C_9H_{10}O_5$	197.04558	4.1609	+0.17	0.829	5	92.31	MSI Level 2
p-Coumaric acid	$C_9H_8O_3$	163.04011	8.4016	+0.26	0.974	5	100.00	MSI Level 2
Epigallocatechin-type flavan-3-ol	$C_{15}H_{14}O_7$	305.06668	3.4386	+0.01	0.900	28	91.32	MSI Level 2
Catechin/epicatechin-type isomer I	$C_{15}H_{14}O_6$	289.07171	5.2805	-0.18	0.895	46	98.99	MSI Level 2
Catechin/epicatechin-type isomer II	$C_{15}H_{14}O_6$	289.07168	6.9350	-0.28	0.902	42	97.25	MSI Level 2
B-type procyanidin dimer I	$C_{30}H_{26}O_{12}$	577.13435	7.2376	-1.39	0.817	11	81.99	MSI Level 2
B-type procyanidin dimer II	$C_{30}H_{26}O_{12}$	577.13537	11.2727	+0.38	0.809	15	96.38	MSI Level 2
Myricetin-O-hexoside	$C_{21}H_{20}O_{13}$	479.08313	9.5673	+0.03	0.855	10	97.55	MSI Level 2
Taxifolin	$C_{15}H_{12}O_7$	303.05104	10.3199	+0.05	0.895	33	93.73	MSI Level 2
Quercetin-O-hexoside I	$C_{21}H_{20}O_{12}$	463.08820	11.2675	+0.00	0.871	7	99.34	MSI Level 2
Quercetin-O-hexoside II	$C_{21}H_{20}O_{12}$	463.08818	11.5736	-0.04	0.821	8	100.00	MSI Level 2
Quercetin-O-glucuronide	$C_{21}H_{18}O_{13}$	477.06751	11.4015	+0.10	0.823	29	94.85	MSI Level 2
Quercetin-O-rhamnoside	$C_{21}H_{20}O_{11}$	447.09325	13.3975	-0.08	0.847	6	94.06	MSI Level 2
Isorhamnetin-O-hexoside	$C_{22}H_{22}O_{12}$	477.10394	13.7446	+0.19	0.846	12	100.00	MSI Level 2
Syringetin-O-hexoside	$C_{23}H_{24}O_{13}$	507.11456	13.8715	+0.29	0.812	14	97.59	MSI Level 2
Piceid (resveratrol-O-hexoside)	$C_{20}H_{22}O_8$	389.12394	13.2384	-0.65	0.938	6	99.10	MSI Level 2
Resveratrol	$C_{14}H_{12}O_3$	227.07133	14.9380	-0.17	0.935	13	98.12	MSI Level 2

RT, retention time; *m*/*z*, mass-to-charge ratio; Δm, mass error between experimental and theoretical $[M{-}H]^-$ exact mass, expressed in ppm; spectral score, similarity between experimental and reference MS/MS spectra; matched MS/MS ions, number of matched fragment ions; explained MS/MS intensity, percentage of experimental fragment-ion intensity accounted for by the library match. In the absence of authentic standards analyzed under identical chromatographic conditions, compounds were reported as putatively annotated (MSI Level 2).
